# Transgressive segregation reveals mechanisms of *Arabidopsis* immunity to *Brassica*-infecting races of white rust (*Albugo candida*)

**DOI:** 10.1073/pnas.1812911116

**Published:** 2019-01-28

**Authors:** Volkan Cevik, Freddy Boutrot, Wiebke Apel, Alexandre Robert-Seilaniantz, Oliver J. Furzer, Amey Redkar, Baptiste Castel, Paula X. Kover, David C. Prince, Eric B. Holub, Jonathan D. G. Jones

**Affiliations:** ^a^The Sainsbury Laboratory, University of East Anglia, Norwich Research Park, NR4 7UH Norwich, United Kingdom;; ^b^The Milner Centre for Evolution, Department of Biology and Biochemistry, University of Bath, BA2 7AY Bath, United Kingdom;; ^c^Institute for Biology, Experimental Biophysics, Humboldt-Universität zu Berlin, 10115 Berlin, Germany;; ^d^Institute for Genetics, Environment and Plant Protection, Agrocampus Ouest, Institut National de la Recherche Agronomique, Universite de Rennes, 35650 Le Rheu, France;; ^e^Department of Biology, University of North Carolina, Chapel Hill, NC 27599;; ^f^Department of Genetics, University of Cordoba, 14071 Cordoba, Spain;; ^g^School of Biological Sciences, University of East Anglia, Norwich Research Park, NR4 7TJ Norwich, United Kingdom;; ^h^Warwick Crop Centre, School of Life Sciences, University of Warwick, CV35 9EF Wellesbourne, United Kingdom

**Keywords:** *Arabidopsis thaliana*, oomycete, *Albugo candida*, nonhost resistance, Brassicaceae

## Abstract

*Arabidopsis thaliana* accessions are universally resistant at the adult leaf stage to white rust (*Albugo candida*) races that infect the crop species *Brassica juncea* and *Brassica oleracea*. We used transgressive segregation in recombinant inbred lines to test if this apparent species-wide (nonhost) resistance in *A. thaliana* is due to natural pyramiding of multiple *Resistance* (*R*) genes. We screened 593 inbred lines from an *Arabidopsis* multiparent advanced generation intercross (MAGIC) mapping population, derived from 19 resistant parental accessions, and identified two transgressive segregants that are susceptible to the pathogen. These were crossed to each MAGIC parent, and analysis of resulting F_2_ progeny followed by positional cloning showed that resistance to an isolate of *A. candida* race 2 (Ac2V) can be explained in each accession by at least one of four genes encoding nucleotide-binding, leucine-rich repeat (NLR) immune receptors. An additional gene was identified that confers resistance to an isolate of *A. candida* race 9 (AcBoT) that infects *B. oleracea*. Thus, effector-triggered immunity conferred by distinct NLR-encoding genes in multiple *A. thaliana* accessions provides species-wide resistance to these crop pathogens.

## Significance

Most plants resist most plant pathogens. Barley resists wheat-infecting powdery mildew races (and vice versa), and both barley and wheat resist potato late blight. Such “nonhost” resistance could result because the pathogen fails to suppress defense or triggers innate immunity due to failure to evade detection. *Albugo candida* causes white rust on most Brassicaceae, and we investigated *Arabidopsis* NHR to *Brassica*-infecting races. Transgressive segregation for resistance in *Arabidopsis* recombinant inbred lines revealed genes encoding nucleotide-binding, leucine-rich repeat (NLR) immune receptors. Some of these NLR-encoding genes confer resistance to white rust in *Brassica* sp. This genetic method thus provides a route to reveal resistance genes for crops, widening the pool from which such genes might be obtained.

Plants and animals are colonized by diverse pathogens and parasites, and their mechanisms of immunity are of broad significance. Plants have two layers of cell-autonomous innate immunity (1–3). Pathogen molecules such as flagellin and chitin are perceived by cell surface pattern recognition receptors (PRRs). Activation of PRRs results in pattern-triggered immunity (PTI) that restricts microbial growth (4, 5). Most plant pathogens translocate pathogenicity proteins, called effectors, into host cells; many of these suppress PTI, facilitating colonization (6–8). Genetic variation for disease resistance within a plant species is often explained by allelic variation in *Resistance* (*R*) genes that encode nucleotide-binding, leucine-rich repeat (NLR) immune receptors. Effector recognition leads to effector-triggered immunity (ETI) (1). Many NLRs carry either Toll/Interleukin-1 receptor/Resistance (TIR-NLRs) or coiled-coil (CC) domains at their N-termini (CC-NLRs) (9–11) and can activate ETI either by directly detecting an effector (12–19) or indirectly through “guarding” host proteins that are modified by effectors (20–22). Unlike CC-NLRs, the function of TIR-NLR proteins requires EDS1 (ENHANCED DISEASE SUSCEPTIBILITY 1), which encodes a lipase-like protein, and forms functional heterodimers in *Arabidopsis* with the related proteins PAD4 (PHYTOALEXIN-DEFICIENT 4) or SAG101 (SENESCENCE-ASSOCIATED GENE 101) (23–25).

Plants are challenged by many potential pathogens but most plants are resistant to most pathogens, and disease is rare. Resistance of a particular plant species against all isolates of a pathogen that can infect other plant species is known as nonhost resistance (NHR) (26). The molecular mechanisms underlying NHR are poorly understood; if all accessions of a species are resistant, genetic analysis of NHR is difficult (27, 28). Conceivably, NHR or species-level resistance could involve PTI (if effectors cannot suppress PTI), ETI (if effectors do not evade detection), and/or other mechanisms (28, 29). Fundamental insights into this question are of broad interest. NHR genes that confer complete immunity in a nonhost might confer resistance in susceptible crops and elevate resistance to important crop diseases.

To investigate NHR, we studied *Albugo candida*, an obligate biotrophic oomycete plant pathogen that causes white blister rust disease in Brassicaceae. In contrast to *A. candida*, *Albugo laibachii* has specialized to cause white rust only on *Arabidopsis* (30). The asexual life cycle of *A. candida* starts with the release of biflagellate motile zoospores from sporangia. Zoospores target host stomata where they encyst and germinate into a germ tube followed by colonization of mesophyll cells by branched hyphae, which also give rise to a specialized feeding structure called an haustorium. Infection culminates in formation of zoosporangia-bearing white pustules that rupture the epidermis; these constitute the visible symptoms of the disease (31). *A. candida* forms many physiological races, each of which specialize on different host species (32–36). Some races of *A. candida* such as Race 2 cause severe annual losses of oilseed mustard (*Brassica juncea*) in India, Canada, and Australia. *Albugo* spp. infection induces a strongly immuno-compromised state in host plants, which can enable avirulent races to colonize and reproduce in the same tissue (37). Sex between different cocolonizing races in the same host could be an important source of new recombinant races (32). Comparative genomics has revealed extensive genetic exchange between races of *A. candida* (34), and this genetic exchange could result in races with novel repertoires of effector alleles that, in turn, might enable colonization of new hosts. Therefore, understanding the underlying mechanism of NHR in different *Brassica* species could inform breeding for resistance to *A. candida*.

Here, we investigate adult plant resistance to *A. candida* Race 2 (Ac2V) in diverse *Arabidopsis thaliana* accessions. While all *Arabidopsis* accessions are resistant to Ac2V, some *A. candida* strains can grow on *Arabidopsis*, but although this pathosystem does not involve NHR to the whole *A. candida* species complex, it is nonetheless instructive. We hypothesized that resistance in *A. thaliana* to Ac2V is due to multiple *R* genes, but the *R* gene repertoire in different *Arabidopsis* accessions might be distinct, creating the potential for transgressive segregation for susceptibility in recombinant inbreds or other segregating progeny from interaccession crosses. We screened a population of “MAGIC” inbred lines (38). These lines result from intercrosses of 19 parents, followed by random intercrossing, and then selfing. These lines have been extensively genotyped (39). We inoculated 593 lines and identified two transgressive segregant inbreds (MAGIC.329 and MAGIC.23) that are susceptible in true leaves to Ac2V. However, none of the MAGIC lines tested, nor the 19 parental accessions, are fully susceptible to Race 9 (AcBoT) collected from *Brassica oleracea*.

We defined three loci that contribute resistance to Ac2V, including a known locus, *White Rust Resistance 4* (*WRR4*) on chromosome 1 (40). *WRR4* carries two paralogs, *WRR4A* and *WRR4B*, that can each confer resistance. We also defined *WRR8* and *WRR9*. To investigate AcBoT resistance in *Arabidopsis*, we intercrossed MAGIC.329 with MAGIC.23. Screening of selfed progeny from this cross revealed fully susceptible plants at a frequency suggesting that resistance in the two parents is conferred by distinct genes. Using RenSeq (Resistance gene Enrichment Sequencing) (41), we identified *WRR12* (previously reported as SOC3) as a gene on chromosome 1 that confers AcBoT resistance (42). These data provide insights into the genetic basis of resistance that restricts pathogen host range and open up a greater subset of the gene pool of crop relatives as a source of genes for crop protection.

## Results

### Identification of Ac2V-Susceptible MAGIC Lines.

**Fig. 1.**
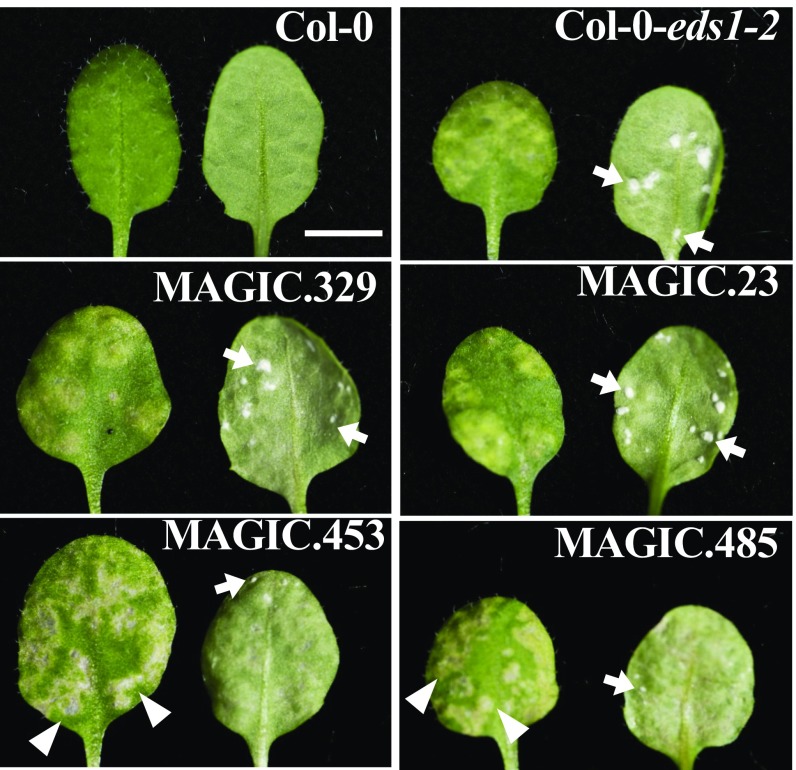
Identification of transgressive segregant MAGIC lines showing different susceptibility to *B. juncea*-infecting *A. candida* race Ac2V. Different levels of susceptibility to Ac2V are observed in an *eds1-2* mutant and in four of 593 MAGIC recombinant inbred lines. Adaxial (*Left*) and abaxial (*Right*) sides of the leaves are presented. Examples of pustules (arrows) and necrotic patches (arrowheads) are indicated. Susceptibility was scored in 4-wk-old plants at 14 dpi. (Scale bars: 3 mm.)

All of 107 previously tested wild-type *Arabidopsis* accessions are resistant to *B. juncea*-infecting *A. candida* race Ac2V, but a Ws-2-*eds1* mutant is susceptible (34). To test if resistance in different *Arabidopsis* accessions is due to distinct resistance gene loci, we evaluated MAGIC lines derived from 19 different *Arabidopsis* accessions (38). We tested Ac2V resistance in 593 MAGIC lines at adult leaf stage with four replicates and identified 10 MAGIC lines that showed either a chlorotic phenotype or different levels of susceptibility. Eight of these 10 lines showed strong chlorotic as well as necrotic patches on infected leaves, although two of these eight lines (MAGIC.453 and MAGIC.485) supported occasional pustule formation (Fig. 1). We regularly observed pustules on the two most susceptible MAGIC lines (MAGIC.23 and MAGIC.329) with Ac2V (Fig. 1). After inoculation with Ac2V, pustules appear 7–10 d after infection (dpi) with MAGIC.329 but later (12–14 dpi) with MAGIC.23 (Fig. 1). However, MAGIC.23 and MAGIC.329 are not as susceptible as Ws-2-*eds1* or Col-*eds1-2* plants.

### Genetic Segregation of Resistance and Susceptibility Phenotypes in F_2_ Progeny Derived from Crosses Between MAGIC Parents and Susceptible MAGIC.329 Line.

Identification of susceptible lines enables genetic analysis of resistance in *Arabidopsis* against Ac2V. We crossed MAGIC.329 with each of the 19 MAGIC parents and selfed F_1_ plants to obtain F_2_ populations. We also analyzed Ws-2 (also known as Ws, Ws-1, Ws-3, and Ws-4, but different from accession Ws-0 that is one of the MAGIC parents) (43) because of its adult plant resistance but seedling susceptibility to Ac2V. All F_1_ progeny were resistant. F_2_ populations were inoculated with Ac2V, and resistance or susceptibility was scored at 14 dpi. We classified F_2_ progeny into three phenotypes: resistant (Green Resistant, GR), partially resistant with chlorosis or necrosis but no pustules (Necrotic-Chlorotic Resistant, NCR), and susceptible, with pustules (Susceptible, S) (Table 1). Segregation ratios ranged from 13R:3S to 255R:1S, suggesting that different *Arabidopsis* accessions carry two to four unlinked *WRR* genes against Ac2V. All tested F_2_ plants from the MAGIC.329 × Wu-0 cross were resistant, suggesting >4 resistance loci.

**Table 1.** Genetic segregation of resistance and susceptibility phenotypes in F_2_ populations between MAGIC.329 and MAGIC parents as well as Ws-2

	Interaction					
F_2_ population	R, GR	R, CNR	S	Expected ratio, (R:S)	No. of loci	*P*
MAGIC.329 x Bur-0	135	24	1	63:1	3	0.34
MAGIC.329 x Can-0	155	41	4	63:1	3	0.61
MAGIC.329 x Col-0	147	10	30	13:3	2*	0.34
MAGIC.329 x Ct-1	140	18	4	63:1	3	0.35
MAGIC.329 x Edi-0	500	16	2	255:1	4	0.98
MAGIC.329 x Hi-0	151	32	23	15:1	2^†^	0.0036
MAGIC.329 x Kn-0	76	79	10	15:1	2	0.92
MAGIC.329 x Ler-0	228	11	16	15:1	2	0.20
MAGIC.329 x Mt-0	154	10	3	63:1	3	0.81
MAGIC.329 x No-0	53	60	1	63:1	3	0.55
MAGIC.329 x Oy-0	206	27	11	15:1	2	0.26
MAGIC.329 x Po-0	74	26	4	15:1	2	0.31
MAGIC.329 x Rsch-4	165	25	32	13:3	2*	0.1
MAGIC.329 x Sf-2	134	115	16	15:1	2	0.07
MAGIC.329 x Tsu-0	223	23	21	15:1	2	0.27
MAGIC.329 x Wil-2	205	69	5	63:1	3	0.75
MAGIC.329 x Ws-0	126	32	11	15:1	2	0.89
MAGIC.329 x Ws-2	170	58	46	13:3	2*	0.40
MAGIC.329 x Wu-0	200	0	0	NT	NT	NT
MAGIC.329 x Zu-0	110	9	2	63:1	3	0.93

GR, green resistant; NCR, necrotic-chlorotic resistant; NT, not tested; *P,* probability value following χ^2^ test**;** R, resistant; S, susceptible.

* One dominant and one recessive gene.

^†^ Two linked genes.

### Most MAGIC Parents Carry Resistance That Maps to the *WRR4* Locus.

The *Arabidopsis WRR4*^Col-0^ gene (*At1g56510*) confers resistance against multiple races of *A. candida* in *Arabidopsis* and in *B. juncea* (33, 40). *WRR4* encodes a TIR-NLR protein. *A. candida* infects by entry of a germ tube into stomata and production of a primary vesicle under an epidermal cell. *WRR4* arrests the development of the pathogen in this epidermal cell, which undergoes a hypersensitive response (HR) (40). As these HR symptoms are not visible macroscopically, we classify this phenotype as GR. We scored susceptible F_2_ individuals using markers at the *WRR4* locus (Dataset S1) and observed cosegregation between Ac2V resistance and *WRR4* for all of the *Arabidopsis* accessions tested except Sf-2 and Wil-2 (*SI Appendix*, Table S1). Cosegregation of Ws-2 resistance with the *WRR4* locus was unexpected, as the *WRR4* gene is absent in Ws-2 (*SI Appendix*, Fig. S1 and Dataset S2), suggesting that at least one more gene at the *WRR4* locus could confer Ac2V resistance.

### Ac2V Resistance in Ws-2 Is Conferred by the *WRR4A* Paralog *WRR4B*.

**Fig. 2.**
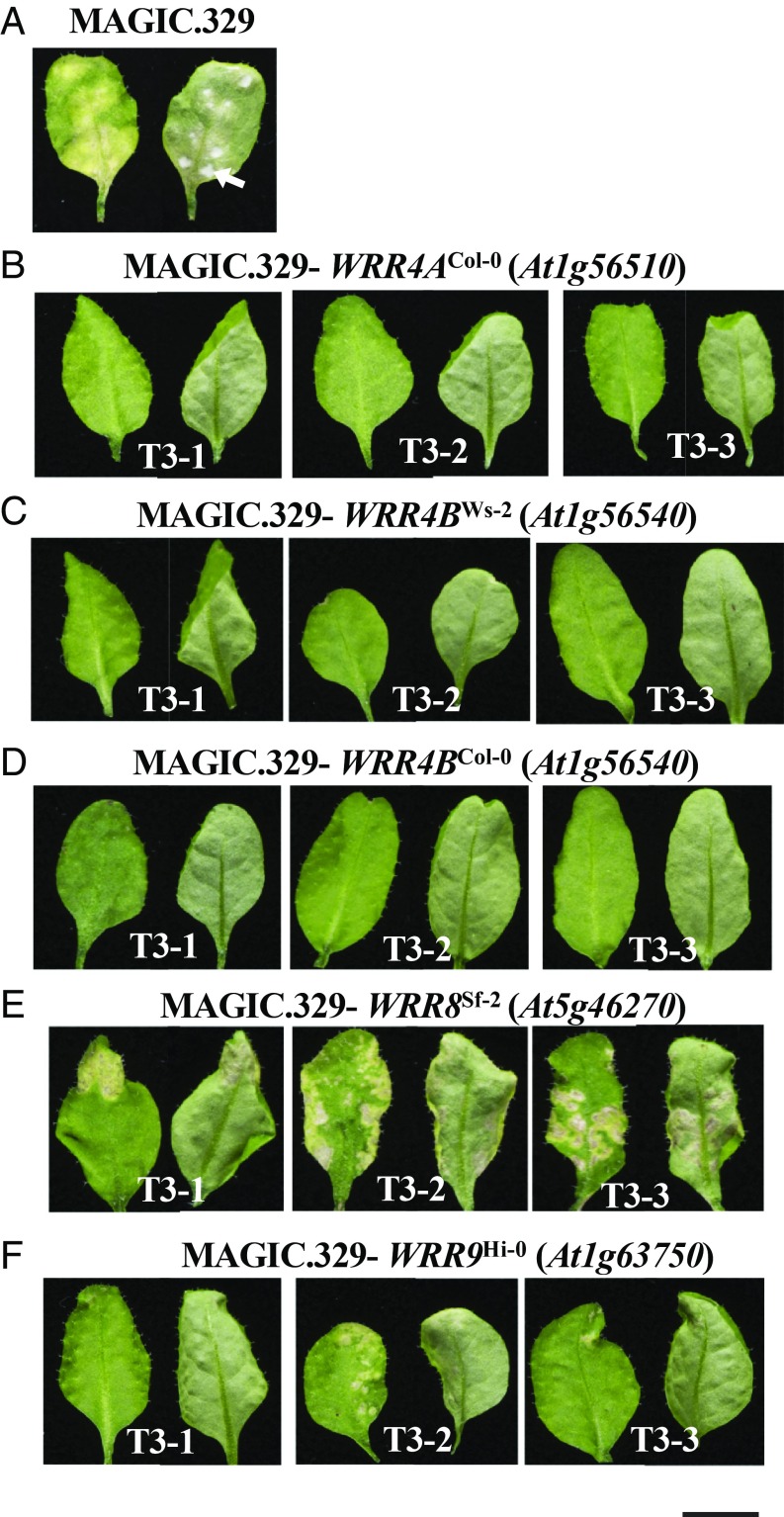
Distinct *WRR* genes confer resistance to Ac2V in the susceptible MAGIC.329 line. (*A*) Nontransformed MAGIC.329 line. (*B*–*F*) Independent homozygous T_3_ MAGIC.329 lines transformed with the genomic clones of *WRR4A*^Col-0^ (*At1g56510*) (*B*), *WRR4B*^Ws-2^ (*At1g56540*) (*C*), *WRR4B*^Col-0^ (*At1g56540*) (*D*), *WRR8*^Sf-2^ (*At5g46270*) (*E*), and *WRR9*^Hi-0^ (*At1g63750*) (*F*). Interaction phenotypes were assayed at 12 dpi. Examples of pustules (arrows) are indicated. (Scale bar: 5 mm.)

Previously, cotyledons of Ws-2 seedlings were found to be susceptible to Ac2V and Ac7V (a *Brassica rapa*-infecting race) but not to AcBoT (a *B. oleracea*-infecting race) (40). However, Ws-2 leaves are fully resistant (GR) to Ac2V and Ac7V. An F_2_ population derived from MAGIC.329 × Ws-2 segregated as 13 GR/NCR: 3 S (*P* = 0.40), suggesting one dominant and one recessive or haplo-insufficient Ac2V resistance gene in Ws-2. All Ac2V-susceptible individuals from the MAGIC.329 × Ws-2 F_2_ lacked the Ws-2 alleles of the markers at the *WRR4* locus. By screening susceptible F_2_ individuals with additional molecular markers (Dataset S1), we found no other loci linked to Ac2V resistance. To improve definition of the resistance locus, we identified 672 Ac2V-susceptible F_2_ plants. We found two recombinants with the molecular marker corresponding to *At1g56040* and only one recombinant with the marker corresponding to *At1g57670*. These markers delineated the locus to ∼397 kb (*SI Appendix*, Fig. S2*A*). *WRR4* maps to this interval in Col-0 but is deleted in Ws-2 (*SI Appendix*, Fig. S1). We therefore cloned two other *WRR4* paralogs *At1g56520* and *At1g56540* from Ws-2 and transformed them into MAGIC.329. For each construct, we tested Ac2V resistance in 48 independent T_1_ plants and in homozygous T_3_ lines. All plants transformed with *At1g56520*^Ws-2^ were susceptible to Ac2V (*SI Appendix*, Fig. S3*A*), but plants with *At1g56540*^Ws-2^ were all resistant (GR) (Fig. 2*C*). We named this gene *WRR4B*. We also cloned the Col-0 allele of *WRR4B*, transformed it into MAGIC.329, and found it also confers resistance to Ac2V (Fig. 2*D*). This suggests that in addition to the broad-spectrum *A. candida* resistance gene *WRR4*^Col-0^ (hereafter *WRR4A*^Col-0^), the *WRR4B* allele of Col-0 functions against Ac2V.

### Ac2V Resistance in Sf-2 Is Conferred by a Resistance Gene, *WRR8*.

Analysis of MAGIC line DNA sequences indicates that the MAGIC.329 WRR4 haplotype derives from Sf-2 (39). As MAGIC.329 is susceptible to Ac2V, this suggests that Sf-2 lacks functional *WRR4A* and *WRR4B* alleles. Screening of susceptible MAGIC.329 × Sf-2 F_2_ progeny confirmed that resistance is unlinked to *WRR4*. We genotyped susceptible F_2_ individuals derived from a MAGIC.329 × Sf-2 cross. A single locus was revealed on chromosome 5 between molecular markers derived from *At5g45400* and *At5g47130* (*SI Appendix*, Fig. S2*B*). Fine mapping using 576 additional susceptible F_2_ individuals revealed an interval between markers derived from *At5g46250* (one recombinant) and *At5g46310* (four recombinants) that carries two TIR-NLR–encoding genes *At5g46260* and *At5g46270* in Col-0. We cloned both genes from *Arabidopsis* accession Sf-2, transformed them into MAGIC.329, inoculated T_1_ plants with Ac2V, and found that transgenic plants carrying *At5g46260*^Sf-2^ were all susceptible (48 of 48), but most plants carrying *At5g46270*^Sf-2^ showed chlorotic resistance (40 of 48) to Ac2V (Fig. 2*E* and *SI Appendix*, Fig. S3*B*). *At5g46270* thus corresponds to *WRR8* in Sf-2.

### Cloning of *WRR9* from *Arabidopsis* Accession Hi-0.

The *WRR4* locus in the *Arabidopsis* accession Hi-0 is linked to Ac2V resistance. Using 352 susceptible F_2_ individuals derived from a MAGIC.329 × Hi-0 cross, we found an additional resistance locus (*WRR9*) on chromosome 1, distinct from *WRR4*. *WRR9* lies between *At1g57670* (one recombinant in 352 plants) and *At1g63820* (one recombinant in 352 plants) (*SI Appendix*, Fig. S2*C*). We thus defined three TIR-NLR *WRR9* candidate genes *At1g63730*, *At1g63740*, and *At1g63750*. We cloned all three genes from Hi-0, transformed into MAGIC.329, and tested T_1_ plants with Ac2V. All of the plants transformed with *At1g63730*^Hi-0^ and *At1g63740*^Hi-0^ were susceptible, but 43 of 48 transgenic T_1_ plants with *At1g63750*^Hi-0^ were resistant to Ac2V (Fig. 2*F*). We infer *WRR9* corresponds to *At1g63750*.

### *WRR4B* but Not *WRR8* and *WRR9* Confer Resistance to Ac2V in *B. juncea*.

**Fig. 3.**
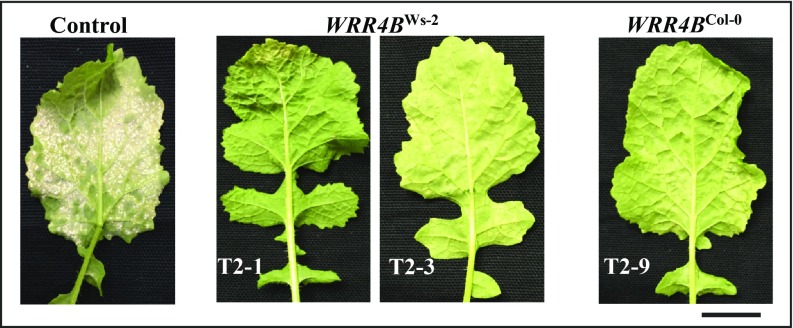
*Arabidopsis WRR* genes provide resistance to *A. candida* race Ac2V in *B. juncea*. Col-0 and Ws-2 alleles of *WRR4B* provide resistance to Ac2V in transgenic *B. juncea*. Nontransgenic control plants and independent T_2_ plants transformed with the indicated *WRR* genes were inoculated with Ac2V. The pictures were taken at 15 dpi. (Scale bar: 10 mm.)

*WRR4A*^Col-0^ confers resistance to two different races of *A. candida* in *B. juncea* and *Brassica napus* (33). We transformed *WRR4B, WRR8*, and *WRR9* into *B. juncea*, obtained two independent transgenic *B. juncea* plants with *WRR4B*^Ws-2^ but only one transgenic plant with the *WRR4B*^Col-0^, and tested T_2_ plants derived from these lines. *WRR4B* transgenic *B. juncea* lines showed green to chlorotic resistance to Ac2V (Fig. 3), resembling the *Arabidopsis* phenotype (Fig. 2 *C* and *D*). We obtained two and four independent transgenic *B. juncea* plants with *WRR8*^Sf-2^ and *WRR9*^Hi-0^, respectively. Following inoculation with Ac2V, the T_2_ plants obtained from these independent transgenic lines were all fully susceptible to the pathogen (*SI Appendix*, Fig. S4), although reverse transcription–PCR (RT-PCR) revealed that *WRR8*^Sf-2^ and *WRR9*^Hi-0^ were expressed in these lines (*SI Appendix*, Fig. S5).

### Transgressive Segregation for AcBoT Susceptibility in a MAGIC.329 × MAGIC.23 F_2_ Reveals WRR12, an Additional TIR-NLR for AcBoT Resistance.

MAGIC.329 and MAGIC.23 are resistant or partially resistant, respectively, to *B. oleracea-*infecting *A. candida* race AcBoT. To identify potential transgressive segregants susceptible to AcBoT, we crossed MAGIC.329 × MAGIC.23 and obtained F_2_ progeny. Inoculation of this F_2_ with AcBoT revealed fully susceptible individuals. The F_2_ population segregated as 15 GR or NCR: 1 S (200GR+34CR:19S) (*P* = 0.41), suggesting a single dominant *WRR* gene is present in each parent. To test if AcBoT-susceptible F_2_ lines are also susceptible to other *Brassica*-infecting *A. candida* races, we obtained F_4_ plants derived from independent susceptible F_2_ lines. We named these plants as “Double MAGIC” (DM) lines. We found that DM lines are also fully susceptible to *A. candida* races Ac2V and Ac7V (*SI Appendix*, Fig. S6).

To identify the underlying genes conferring resistance to AcBoT in MAGIC.329 and MAGIC.23, we collected ∼200 fully susceptible F_2_ individuals following AcBoT inoculation. To accelerate the cloning, we conducted RenSeq (41) on DNA of the resistant parents MAGIC.329 and MAGIC.23 as well as bulked susceptible DNA (BS) obtained from the fully susceptible F_2_ individuals. MiSeq reads obtained from the parents and from BS were used to identify polymorphisms and linkage by mapping the reads to the Col-0 reference genome. This revealed a single locus where the resistance gene from MAGIC.329 is located (*SI Appendix*, Table S2). We named this gene *WRR12* and found that, in MAGIC.329, this genomic region was introgressed from Ler-0, whereas the nonfunctional allele in MAGIC.23 was introgressed from Wu-0. We found no additional locus linked to the resistance in MAGIC.23, suggesting that its partial resistance could be multigenic. Three genes within the *WRR12* locus cosegregate with resistance (*SI Appendix*, Table S2): the TIR-NLR gene *At1g17600* and TIR-NB–only genes *At1g17610* and *At1g17615* (*SI Appendix*, Fig. S7). *At1g17600* was previously designated *SUSA1* or *SOC3* and implicated in cold-induced activation of defense by an allele of *At1g17610* (CHS1) (42, 44, 45). Recently, SOC3/CHS1 was proposed to “guard” an immune-regulating E3 ligase SAUL1 (46). TN2 (*At1g17615*) was reported to be required for the enhanced disease resistance phenotype in *exo70B1* mutant *Arabidopsis* plants (47).

**Fig. 4.**
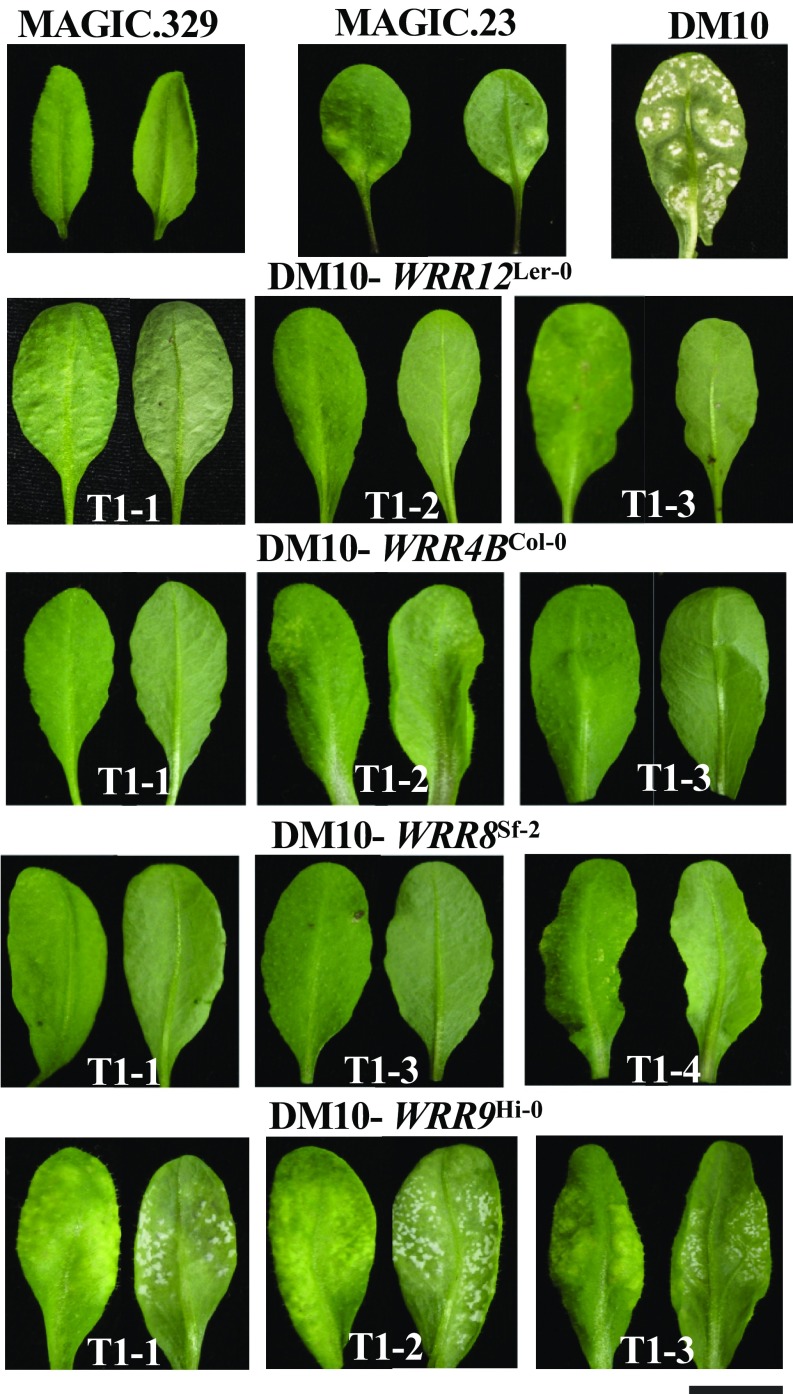
*WRR12*^Ler-0^, *WRR4B*^Col-0^, *WRR8*^Sf-2^, but not *WRR9*^Hi-0^ confer resistance to *B. oleracea*-infecting *A. candida* race AcBoT in *Arabidopsis*. MAGIC.329 and MAGIC.23 are resistant or partially resistant, respectively, to AcBoT. DM10 lines were transformed with *WRR12*^Ler-0^ (*At1g17600*), *WRR4B*^Col-0^, *WRR8*^Sf-2^, and *WRR9*^Hi-0^ and interaction phenotypes were assayed in independent T_1_ plants at 20 dpi. (Scale bar: 10 mm.)

The *At1g17600* allele from Wu-0 in MAGIC.23 (and only this allele; Dataset S2) carries a ∼4-kb transposon insertion (*SI Appendix*, Fig. S7 and Dataset S2), suggesting that it is nonfunctional, and that the Ler-0 allele in MAGIC.329 is a strong candidate for *WRR12-*mediated resistance. We cloned *At1g17600* from MAGIC.329 and transformed into line DM10, one of the DM lines. Independent T_1_ transgenic plants were screened with *A. candida* race AcBoT. All 24 T_1_ transgenic DM10 plants were resistant to AcBoT. This suggests that *At1g17600*^Ler-0^ corresponds to *WRR12* (Fig. 4). We also transformed *WRR4B*^Col-0^, *WRR8*^Sf-2^, and *WRR8*^Hi-0^ into DM10 to determine if these genes confer resistance to AcBoT in *Arabidopsis*. We found all *WRR4B*^Col-0^ transgenic T_1_ plants (eight of eight) were resistant to AcBoT, while seven of eight *WRR8*^Sf-2^ transgenic plants showed resistance to the pathogen. In contrast, *WRR9*^Hi-0^ transgenic DM10 lines (nine of nine) were fully susceptible to AcBoT (Fig. 4).

In addition, we transformed *B. oleracea* DH1012 with *WRR4A*^Col-0^, *WRR4B*^Col-0^, and *WRR4B*^Ws-2^, as well as *At1g56520*^Col-0^ as a negative control and inoculated independent T_1_ transgenic *B. oleracea* lines with AcBoT. T_1_ transgenic plants with *WRR4A*^Col-0^ (15 of 16), *WRR4B*^Ws-2^ (13 of 19), and *WRR4B*^Col-0^ (two of two) were fully resistant to AcBoT, whereas transgenic plants with *At1g56520*^Col-0^ (four of four) were fully susceptible (*SI Appendix*, Fig. S8).

### *WRR* Gene Haplotypes in MAGIC Parents.

To determine the distribution and sequence variation of *WRR4A*, *WRR4B*, *WRR8*, *WRR9*, and *WRR12* genes, the MAGIC parents as well as Ws-2 were sequenced using SMRT RenSeq (48). The sequences of the *WRR* alleles from each accession were identified by blastn (49) against the SMRT RenSeq assemblies. Blastn hits showing less than 95% identity were not considered to be alleles of the *WRR* genes. We used the Augustus gene prediction server (50) to obtain predicted protein sequences of the *WRR* alleles. We identified *WRR4A* alleles in all MAGIC parent accessions except Ws-2, Edi-0, and No-0 (Dataset S2). We also identified *WRR4B* alleles in all *Arabidopsis* accessions except Tsu-0 in the RenSeq assemblies. Both Sf-2 and Wil-2 (source of the *WRR4* haplotypes in MAGIC.329 and MAGIC.23, respectively) lack functional *WRR4A* and *WRR4B* genes. We identified RenSeq assemblies for *WRR4A* and *WRR4B* in both *Arabidopsis* accessions, and the lack of functional *WRR4A* and *WRR4B* in Sf-2 and Wil-2 is not due to deletion (Dataset S2). Although the Sf-2 *WRR4A* region was not clearly resolved in the de novo assembly, by aligning the RenSeq reads to the Col-0 genome, we confirmed a single base deletion, also observed in the *Arabidopsis* 1001 genomes browser, at nucleotide position 177 that results in an early stop codon, explaining why the Sf-2 *WRR4A* allele is nonfunctional.

Blastn analysis revealed that all of the *Arabidopsis* accessions contain *WRR8* and *WRR9* alleles except for *WRR9* in Ler-0. However, as for the *WRR4A* and *WRR4B* alleles, some of the assemblies did not cover full-length *WRR8* and *WRR9*. This is most likely due to partial SMRT RenSeq assemblies or incomplete capture.

We also identified *WRR12* alleles in MAGIC parents and Ws-2. All lines carried an apparently functional allele, except for Wu-0.

## Discussion

NHR in one plant species can be defined as complete resistance to pathogens that infect another species (26). Multiple mechanisms, such as preformed antimicrobial metabolites, and induced defenses such as PTI and ETI, could contribute to NHR (51, 52). A better understanding of the mechanisms of NHR could reveal additional genes that confer resistance in crops to plant pathogens.

We investigated NHR in *Arabidopsis* against *Brassica*-infecting *A. candida* races. All *Arabidopsis* accessions tested are resistant to *B. juncea*-infecting race Ac2V, *B. rapa*-infecting race Ac7V, and *B. oleracea*-infecting race AcBoT (ref. 34, this study). However, we found that both Col-0-*eds1-2* (53) and Ws-2-*eds1* (34) are susceptible to all three *A. candida* races, suggesting that NHR to these races might involve TIR-NLR genes (23). We further hypothesized that resistance in different *Arabidopsis* accessions could be mediated by distinct resistance genes. Therefore, we screened MAGIC lines derived from 19 different *Arabidopsis* parents (38) and identified transgressive segregant lines that are susceptible to Ac2V. These susceptible plants enabled us to perform genetic analysis to identify resistance genes in multiple *Arabidopsis* accessions.

We defined three *WRR* (*WRR4B*^Col-0^, *WRR8*^Sf-2^, and *WRR9*^Hi-0^) genes against Ac2V, and a gene, *WRR12* (*SOC3*), conferring NHR to AcBoT, in addition to the previously identified broad spectrum resistance gene *WRR4A*^Col-0^. Other investigations have revealed additional *WRR* genes, but we focus in this paper on resistances at the *WRR4*, *8*, *9*, and *12* loci. A point mutation in *At1g17610*, the neighboring gene of *WRR12* encoding a TIR-NB protein, results in *chilling sensitive 1* (*CHS1*), with an autoactive defense phenotype (44). This phenotype could be suppressed by mutations in *WRR12*, which was therefore named *suppressor of chilling sensitive 1–3* (*SOC3*). SOC3 and CHS1 can associate physically (42).

A phylogenetic analysis using an alignment of the NB-ARC region of TNLs in *Arabidopsis* accession Col-0 reveals that WRR4, WRR4B, and WRR9 are monophyletic, suggesting they shared a more recent common ancestor than with WRR8 (*SI Appendix*, Fig. S9). This analysis also reveals that WRR12 and CHS1 are located in neighboring expanded clades, many members of which are part of divergently transcribed pairs in the Col-0 genome (*SI Appendix*, Fig. S9). This suggests that multiple duplications of an ancestral WRR12/CHS1 pair occurred, similar to the expansion that occurred of RPS4/RRS1-like pairs (refs. 54 and 55 and *SI Appendix*, Fig. S9).

Neither *WRR8* nor *WRR9* confer resistance to Ac2V in *B*. *juncea*, although these genes confer resistance in *Arabidopsis*. *WRR8* also confers resistance to AcBoT in *Arabidopsis*. This could be due to the fact that WRR8- and WRR9-mediated resistance involves a guardee or decoy that is present in *Arabidopsis* but absent or divergent in *Brassica* sp. Indeed, recent publications show that *WRR12/SOC3* and *CHS1* form a gene pair and that WRR12/SOC3, together with CHS1, monitors the homeostasis of E3 ligase SAUL1, a potential guardee that we hypothesize might be targeted by *A. candida* effector(s) (42, 46).

F_2_ individuals from crosses between MAGIC.329 and Col-0, Rsch-4, or Ws-2 segregated at a ratio of 13:3, suggesting one dominant and one recessive or haplo-insufficient gene. Identification of a second resistance locus in these F_2_s will require genotyping fully resistant individuals that lack resistant *WRR4* haplotypes. Crosses between MAGIC.329 and Oy-0 or Sf-2 show a 15:1 segregation in the F_2_, suggesting two independent dominant resistance loci, but genotyping susceptible plants revealed only one locus. How many more *WRR* genes might there be in *Arabidopsis*? All F_2_ individuals resulting from selfing the F_1_ between MAGIC.329 × Wu-0 are resistant, suggesting that Wu-0 likely contains >4 resistance loci, so additional loci for resistance to Ac2V and AcBoT likely remain to be discovered.

Our data suggest that *Arabidopsis* NHR against *Brassica*-infecting *A. candida* races is primarily mediated via ETI, consistent with the expectation that ETI is more likely to contribute to NHR if there is a close evolutionary relationship between the host and nonhost plant species (29). ETI may contribute to NHR in other plant pathosystems. For example, various RxLR effectors from *Phytophthora infestans* trigger a HR in the nonhost pepper (56). NHR to *P. capsici* in various *Nicotiana* species likely involves PcAvr3a1 effector recognition (57). *Pseudomonas syringae* AvrRps4 homologs (HopK1_DC3000_ and AvrRps4_Pph1448A_) trigger HR in lettuce, and this HR phenotype cosegregates with a NLR locus RGC4 (58). NHR against wheat stripe rust (*Puccinia striiformis* f. sp. *tritici*) in barley or in *Brachypodium distachyon* was mapped to *Rps6* or *Yrr2* loci, respectively. Both intervals were shown to contain NLR genes, suggesting that NLRs may contribute to NHR against wheat stripe rust in barley and *B. distachyon* (59–61). Furthermore, nonhost resistance to *Lolium* and *Avena* isolates of *Pyricularia oryzae* in wheat was shown to be mediated by two resistance genes, *Rwt3* and *Rwt4*, and the emergence of wheat blast was attributed to a host jump as a result of widespread growth of *rwt3* wheat (62).

NLR-encoding resistance genes recognize pathogen effectors. When *A. candida* races of Ac2V and Ac7V were intercrossed, and F_2_ individuals obtained and inoculated on *B. rapa* (host for Ac7V but nonhost for Ac2V), a segregation ratio of three avirulent to one virulent was obtained. This supports the hypothesis that resistance to Ac2V in *B. rapa* involves resistance gene-dependent recognition of an Ac2V effector allele that is absent from or different in Ac7V (63).

Specific races of *A. candida*, usually considered a generalist pathogen, colonize a particular host species (34). Why might resistance genes in nonhost plants recognize effectors from nonadapted pathogens? Conceivably, host and nonhost plants share a common ancestor that was a host for the pathogen (56). Our data suggest that host/race specificity of *A. candida* is determined by the NLR repertoire of the host plant and the recognized effectors of the pathogen race, rather than host compatibility factors. Therefore, some of the NLRs recognizing specific races or multiple races are maintained in different Brassicaceae species. This, in turn, provides an excellent resource to identify *WRR* genes for different *Brassica* species. In summary, by using transgressive segregation to reveal susceptible lines, we were able to reveal genes that underpin resistance in *Arabidopsis* to *Brassica*-infecting *A. candida* races and show that some of these genes might be useful for elevating crop disease resistance. This strategy could also be applied to identify useful new resistance genes in other crop relatives that show NHR to crop-adapted pathogen races.

## Materials and Methods

All *Arabidopsis* accessions used in this study were obtained from the Nottingham *Arabidopsis* Stock Centre. Col-0-*eds1-2* and Ws-2-*eds1* were described in refs. 32 and 48. MAGIC lines were described in ref. 38. *Arabidopsis* seeds were sown on Scotts Levington F2 compost (Scotts) and vernalized for 1 wk at 5–6 °C. Seedlings were subsequently grown in a controlled environment room (CER) with a 10-h day and a 14-h night photoperiod and at a constant temperature of 22 °C for 2 wk and then pricked-out into “Arabidopsis mix” [Scotts Levington F2 compost-grit (6:1, vol/vol), 0.03% (m/v) Intercept insecticide] and returned to the CER. *B. juncea* seeds were sown on Scotts Levington F2 compost. Seedlings were subsequently grown in a controlled environment room (CER) with a 10-h day and a 14-h night photoperiod and at a constant temperature of 22 °C for 1 wk and then pricked-out into Arabidopsis mix and returned to the CER. Detailed information is provided in *SI Appendix*, *Supplementary Materials and Methods*.

## Supplementary Material

Supplementary File

Supplementary File

Supplementary File

Supplementary File
