# Supplementary material for: Transgressive segregation reveals mechanisms of Arabidopsis immunity to Brassica-infecting races of white rust (Albugo candida)
Source: Proc Natl Acad Sci U S A. 2019 Jan 28;116(7):2767–73. doi: 10.1073/pnas.1812911116 (PMC6377460; doi:10.1073/pnas.1812911116)
Supplement: Supplementary File [file pnas.1812911116.sapp.pdf]

Supporting Information for

Transgressive segregation reveals mechanisms of *Arabidopsis* immunity to *Brassica*-infecting races of white rust (*Albugo candida)*

Volkan Cevik[1,2], Freddy Boutrot[1], Wiebke Apel[1,3], Alexandre Robert-Seilaniantz[1,4], Oliver J. Furzer[1,5], Amey Redkar[1,6], Baptiste Castel[1], Paula X. Kover[2], David C. Prince[1,7], Eric B. Holub[8] and Jonathan D G Jones[1]*

1. The Sainsbury Laboratory, University of East Anglia, Norwich Research Park, Colney Lane, Norwich, NR4 7UH, United Kingdom
2. The Milner Centre for Evolution, Department of Biology and Biochemistry, University of Bath, Bath, BA2 7AY, United Kingdom
3. Institute for Biology, Experimental Biophysics, Humboldt-Universität zu Berlin, 10115 Berlin, Germany
4. Institute for Genetics, Environment and Plant Protection, Agrocampus Ouest, Institut National de la Recherche Agronomique, Universite de Rennes, 35650 Le Rheu, France
5. Department of Biology, University of North Carolina, Chapel Hill, Chapel Hill, North Carolina, NC 27599, United States of America
6. Department of Genetics, University of Cordoba, 14071 Cordoba, Spain
7. School of Biological Sciences, University of East Anglia, Norwich Research Park, Norwich, NR4 7TJ, United Kingdom
8. Warwick Crop Centre, School of Life Sciences, University of Warwick, Wellesbourne, CV35 9EF, United Kingdom

* Corresponding author: jonathan.jones@tsl.ac.uk

This PDF file includes:

Supplementary Materials and Methods

Figs. S1 to S9

Table S1 to S3

Captions for datasets S1 to S3

Other supplementary materials for this manuscript include the following:

Datasets S1 to S3

www.pnas.org/cgi/doi/10.1073/pnas.1812911116

Supplementary Materials and Methods

Pathogen strains and maintenance

Albugo candida races Ac2V, Ac7V and AcBoT were propagated as follows: zoosporangia from plants inoculated 14 days earlier were suspended in cold water and incubated on ice for 30 min. The spore suspension was then sprayed on plants using a spray gun, and plants were incubated in a cold room (5°C) in the dark over-night. Infected plants were kept under 10 hrs light and 14 hrs dark cycles with a 21°C day and 14°C night temperature. *Albugo candida* race Ac2V was maintained on *Brassica juncea* cultivar Burgonde, Ac7V on *B. rapa* cultivar Just Right and AcBoT on *Brassica oleracea* DH1012 (1). Pathogen infection assays were carried out using two weeks old *Arabidopsis* plants and the inoculations were carried out as described above.

DNA Extraction

For both genetic mapping and RenSeq, gDNA was extracted from young leaves using the DNeasy Plant Mini Kit (Qiagen, Hilden, Germany) following the manufacturer's protocol.

Genetic Mapping

To identify linkage with the *WRR* locus of interest, DNA extracted from fully susceptible F_2 plants derived from the crosses between MAGIC.329 and the indicated *Arabidopsis* accession were used for genotyping with the molecular markers located on different *Arabidopsis* chromosome arms that show polymorphism between MAGIC.329 and the indicated *Arabidopsis* parent. Molecular markers and corresponding primer sequences are in Dataset S1. Once a linkage established, further molecular markers were developed and used to delineate the *WRR* locus of interest using additional fully susceptible F_2 plants.

Cloning of *WRR* genes

Genomic fragment of *WRR4A*$^{Col\text{-}0}$ was amplified from *Arabidopsis* accession Col-0 with Platinum Pfx DNA Polymerase (Life Technologies, Carlsbad, CA, USA) using the specific primers given in Table S3. Following digestion with *BamH*I (New England Biolabs, Inc., Ipswich, MA, USA) , this genomic fragment was cloned in to *BamH*I and *Sma*I (New England Biolabs, Inc., Ipswich, MA, USA) digested pCambia2300 vector. *WRR4B* alleles as well as its paralog *At1g56520* were amplified from *Arabidopsis* accessions Col-0 and Ws-2 with Platinum Pfx DNA Polymerase (Life Technologies, Carlsbad, CA, USA) as multiple fragments using the primers containing a *Bsa*I recognition site (Table S3). Using the Golden Gate assembly (2), these fragments were assembled into full-length genes and cloned into the binary vector pICH86966. *WRR8*$^{Sf\text{-}2}$ and its paralog *At5g46260* from Sf-2, *WRR9*$^{Hi\text{-}0}$ and its paralogs *At1g63730* and *At1g63740* from Hi-0 as well as *WRR12*$^{Ler\text{-}0}$ allele from MAGIC.329 were PCR amplified with Kapa Hifi Uracil+ (Roche) using primers supplemented with specific 5' and 3' extensions (Table S3) to make them compatible with custom USER (Uracil-Specific Excision Reagent) expression vectors (3). 30 ng of purified PCR product was then hybridized with 30 ng *Pac*I and *Nt.B*bvCI (New England Biolabs, Inc., Ipswich, MA, USA) digested USER vector pICSLUS0002 in the presence of 1 µL of USER enzyme mix (New England Biolabs, Inc., MA, USA). All constructs were verified by DNA sequencing. Plasmids containing the candidate genes were then transformed into *Agrobacterium tumefaciens* strain AGL1. We submitted novel *WRR* gene sequences to NCBI GenBank (4) (accession numbers): *WRR4B*$^{Ws\text{-}2}$ (MK034466), *WRR4B*$^{Col\text{-}0}$ (MK034465), *WRR8*$^{Sf\text{-}2}$ (MK034463), *WRR9*$^{Hi\text{-}0}$ (MK034464) and *WRR12*$^{Ler\text{-}0}$ (MK034462).

Plant Transformation

Arabidopsis MAGIC.329, DM4, DM6, DM9 and DM10 plants were transformed using floral dipping method (5). Following transformation, T_1 transgenic plants were selected on

selective MS media. T_2 seeds obtained were then sown on selective MS media, and lines with single T-DNA insertions were identified. For *Brassica* transformation, seeds were first sterilized for 2 minutes in 70 % ethanol followed by a solution containing 15% sodium hypochlorite (Bleach) and a few drops of Tween 20 for 10 minutes. Seeds were then rinsed with sterile water for 4 times. Seeds were dried with sterile filter paper and placed onto petri dishes with germination media and placed in to a growth chamber (at 22°C under 16 hrs light) for 4 days. Explants were prepared by removing the hypocotyls, being careful to not take the cotyledons or radicle. Explants were then inoculated with Agrobacterium suspension (a final OD of 0.8 with 2 mg/L BAP, 0.5 mg/L NAA, 150 μM acetosyringone) for 10 mins. They were then placed on co-cultivation media containing 2 mg/L BAP, 0.5 mg/L NAA, 150 μM acetosyringone and 5 mg/L $AgNO_3$ and incubated for 3 days at 22°C and 16 hrs light. The explants were then transferred onto regeneration media containing 2 mg/L BAP, 0.5 mg/L NAA, 5 mg/L AgNO3 and 300 mg/L Timentin and incubated for 7 days at 22°C under 16 hrs light. After 7 days, explants were moved to regeneration media containing 2mg/L BAP, 0.5mg/L NAA, 5 mg/L $AgNO_3$, 300 mg/L Timentin and 25 mg/L kanamycin and incubated for 14 days and subbing was carried out onto fresh media every 14 days until shoots appeared. Antibiotic resistant shoots were removed from explants and placed in sterilin jars containing rooting media with 300 mg/L Timentin, 25 mg/L kanamycin and 0.5 mg/L IBA.

RNA extraction, cDNA synthesis and RT-PCR

To determine the expression of *WRR8*$^{\text{Sf-2}}$ and *WRR9*$^{\text{Hi-0}}$ in transgenic *Brassica juncea*, total RNA was extracted using the TRI-reagent (Sigma- Aldrich, MO, USA) and Directzol RNA Mini-prep kit (Zymo Research, CA, USA), following manufacturer's recommendations. First-strand cDNA was made with Invitrogen Superscript III Reverse Transcriptase. The obtained cDNA was used as template for RT-PCR with specific primers listed in Table S3.

Capture library design for Bassicaceae and Resistance gene Enrichment Sequencing

For the Illumina MiSeq RenSeq experiment, 2 µg of gDNA from Bulk Susceptible (BS) MAGIC329 and MAGIC23 were fragmented with the Covaris sonicator (Covaris Inc. MA, USA) using preset 1 kb settings. Fragments were size selected for fragments longer than 500 bp using AmPureXP beads (Beckman Coulter, CA,USA) with 1:0.55 ratio of sheared DNA to AMPureXP beads. Illumina MiSeq gDNA libraries were prepared using the NEBNext Ultra DNA Library Prep Kit for Illumina (New England Biolabs, Inc., Ipswich, MA, USA) following Manufacturer's instructions. Target capture was carried out using a custom MYcroarray MYbaits kit (MI, USA) and the corresponding protocol (6). 20,000 synthetic 120 nt biotinylated RNA probes (*baits*), complementary to 736 NLR-encoding genes from *Arabidopsis thaliana*, *Arabidopsis lyrata*, *Brassica rapa*, *Aethionema arabicum* and *Eutrema parvulum* were synthesized by MYcroarray and used to perform capture (Dataset S3.).

10 µL of enriched library was PCR amplified (KAPA HiFi enzyme) to a quantity of 1 µg in 50 µL reaction volumes using Illumina P5 and P7 primers. MiSeq 250-bp paired end (PE) sequencing was carried out at Earlham Institute, Norwich Research Park, UK. The reads were aligned to the *Arabidopsis* reference genome (TAIR10) using Burrow Wheelers Aligner BWA version 0.7.4 (7) was used with the BWA-MEM algorithm. SAMtools (8) version 0.1.19 was used to generate BAM files. Polymorphisms were visualized and confirmed in BAM files with the Integrative Genomics Viewer (9). To obtain linked polymorphisms, we determined the SNPs between resistant and susceptible parents and identified SNPs with >95% allele frequencies in BS derived the susceptible parent. Illumina reads were submitted under ENA (10) project PRJEB26457.

Extraction of sequences of *WRR* alleles from SMRT RenSeq assemblies

Eighteen MAGIC parents as well as Ws-2 were sequenced using the SMRT RenSeq technology (11) as part of a collaborative effort.

http://ftp.tuebingen.mpg.de/ebio/alkeller/pan_NLRome/. We also independently sequenced one of the MAGIC parents Can-0 using the SMRT RenSeq technology and assembled using CANU version 1.3 (12). Full sequencing, assembly statistics and associated quality control steps for the eighteen MAGIC parents and Ws-2 will be published in an upcoming submission. Reads and assemblies for these accessions can be found online at http://ftp.tuebingen.mpg.de/ebio/alkeller/pan_NLRome/. SMRT RenSeq sequence reads for Can-0 were submitted under ENA project PRJEB26457. To discover putative *WRR* alleles from each accession, the genomic sequence of each respective *WRR* gene was queried using the blastn command (13) (megablast) against the assembly of SMRT RenSeq reads. The highest ranked hits from these searches were compiled. Hits of less than 95% identity and much shorter than the query sequence were considered non-orthologous loci and are marked in grey in Dataset S2. To obtain predicted protein sequences the assemblies were submitted to the Augustus gene prediction server (14) using the *Arabidopsis thaliana* species parameters. Blastp was used to query the predicted amino acid sequence encoded by each WRR gene against a combined database of all the predicted proteins in the MAGIC founders and Ws-2.

Phylogeny construction

In order to construct the phylogeny of *Arabidopsis thaliana* proteins containing TIR and NB domains, TIR-domain containing proteins were identified from the Araport11 Col-0 annotation using NLR-parser (15). Additionally, APAF-1 *(Homo sapiens)* and L6 (*Linum usitatissimum*) were chosen as outgroups. Where NB domains were detected, these were extracted and a core region of ~200 amino acids was aligned using MUSCLE (16) and phylogenies constructed using the WAG +F model with 5 discrete gamma categories and a bootstrap (100x) test of phylogeny (17), both within the MEGA6 suite (18). Resulting phylogenies were prepared using figtree v1.4.3 (http://tree.bio.ed.ac.uk/software/figtree/).

References

1. Sparrow PA, Dale PJ, & Irwin JA (2006) *Brassica oleracea*. *Methods Mol Biol* 343:417-426.
2. Engler C & Marillonnet S (2011) Generation of families of construct variants using golden gate shuffling. *Methods Mol Biol* 729:167-181.
3. Nour-Eldin HH, Geu-Flores F, & Halkier BA (2010) USER cloning and USER fusion: the ideal cloning techniques for small and big laboratories. *Methods Mol Biol* 643:185-200.
4. Benson DA*, et al.* (2018) GenBank. *Nucleic Acids Res* 46(D1):D41-D47.
5. Clough SJ & Bent AF (1998) Floral dip: a simplified method for Agrobacterium-mediated transformation of *Arabidopsis thaliana*. *Plant J* 16(6):735-743.
6. Jupe F*, et al.* (2013) Resistance gene enrichment sequencing (RenSeq) enables reannotation of the NB-LRR gene family from sequenced plant genomes and rapid mapping of resistance loci in segregating populations. *Plant J* 76(3):530-544.
7. Li H & Durbin R (2009) Fast and accurate short read alignment with Burrows-Wheeler transform. *Bioinformatics* 25(14):1754-1760.
8. Li H*, et al.* (2009) The Sequence Alignment/Map format and SAMtools. *Bioinformatics* 25(16):2078-2079.
9. Thorvaldsdottir H, Robinson JT, & Mesirov JP (2013) Integrative Genomics Viewer (IGV): high-performance genomics data visualization and exploration. *Brief Bioinform* 14(2):178-192.
10. Silvester N*, et al.* (2018) The European Nucleotide Archive in 2017. *Nucleic Acids Res* 46(D1):D36-D40.
11. Witek K*, et al.* (2016) Accelerated cloning of a potato late blight-resistance gene using RenSeq and SMRT sequencing. *Nat Biotechnol* 34(6):656-660.
12. Koren S*, et al.* (2017) Canu: scalable and accurate long-read assembly via adaptive k-mer weighting and repeat separation. *Genome Res* 27(5):722-736.
13. Camacho C*, et al.* (2009) BLAST+: architecture and applications. *BMC Bioinformatics* 10:421.
14. Hoff KJ & Stanke M (2013) WebAUGUSTUS--a web service for training AUGUSTUS and predicting genes in eukaryotes. *Nucleic Acids Res* 41(Web Server issue):W123-128.
15. Steuernagel B, Jupe F, Witek K, Jones JD, & Wulff BB (2015) NLR-parser: rapid annotation of plant NLR complements. *Bioinformatics* 31(10):1665-1667.
16. Edgar RC (2004) MUSCLE: a multiple sequence alignment method with reduced time and space complexity. *BMC Bioinformatics* 5:113.
17. Whelan S & Goldman N (2001) A general empirical model of protein evolution derived from multiple protein families using a maximum-likelihood approach. *Mol Biol Evol* 18(5):691-699.
18. Tamura K, Stecher G, Peterson D, Filipski A, & Kumar S (2013) MEGA6: Molecular Evolutionary Genetics Analysis version 6.0. *Mol Biol Evol* 30(12):2725-2729.

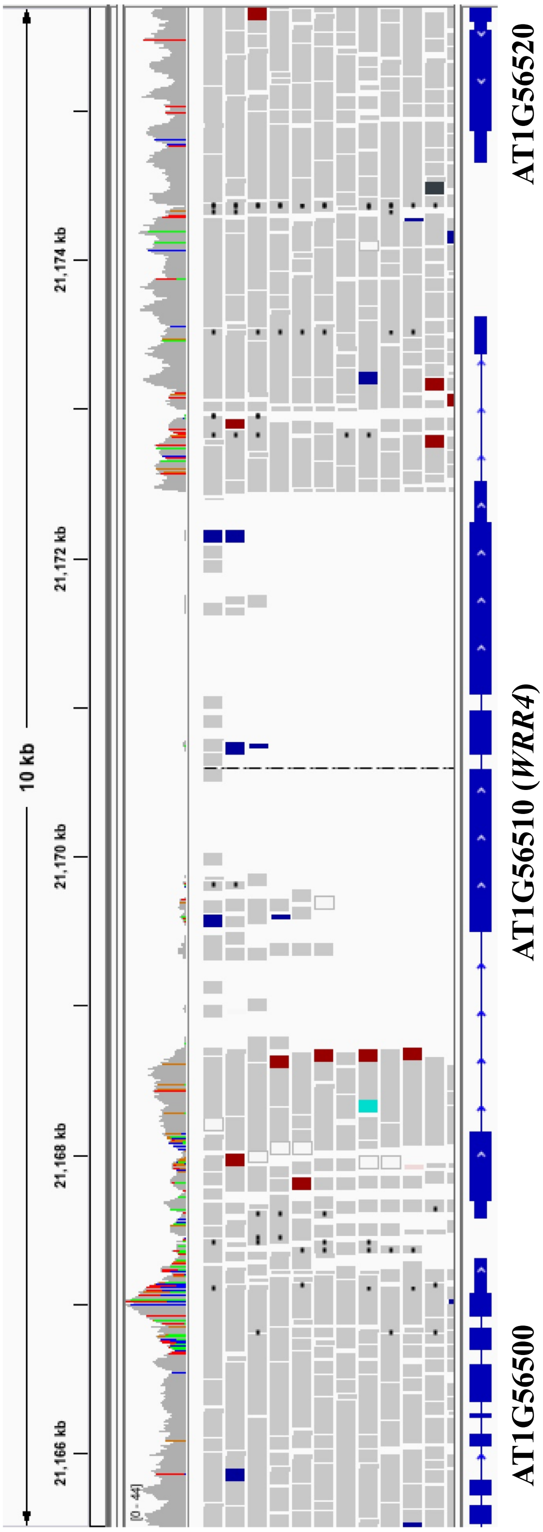

Fig S1. Integrated Genome View following mapping the Ws-2 whole genome Illumina reads to Col-0 TAIR10 reference. *At1g56510* encodes for the WRR4 resistance protein

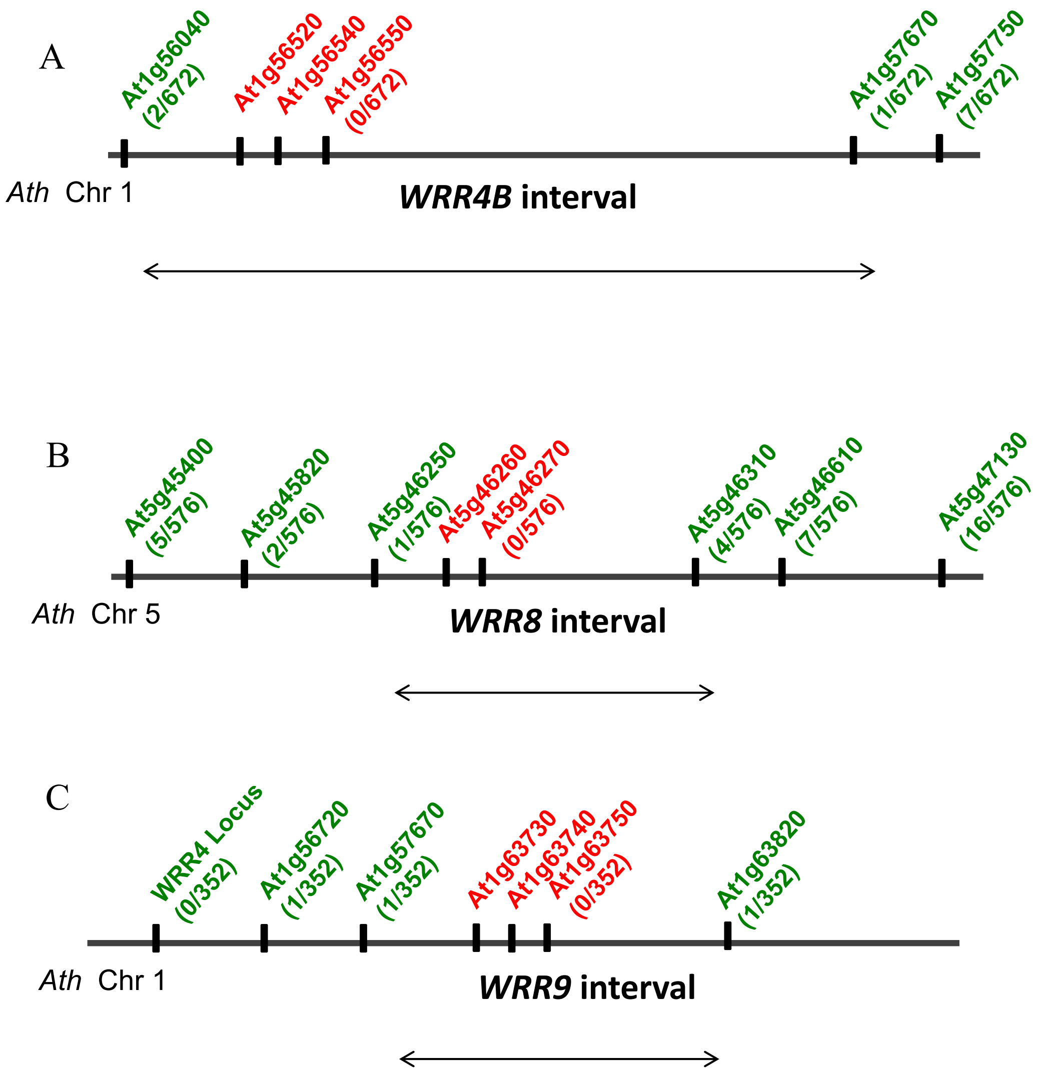

Fig S2. Fine mapping of the *WRR4B*$^{\text{Ws-2}}$, *WRR8*$^{\text{Sf-2}}$ and *WRR9*$^{\text{Hi-0}}$ genes against *A. candida* race Ac2V. (A) Total of 672 susceptible F_2 individuals were screened with the molecular markers in order to delineate the *WRR4B*$^{\text{Ws-2}}$ locus. (B) 576 susceptible individuals were used to narrow the *WRR8*$^{\text{Sf-2}}$ locus. (C) The *WRR9*$^{\text{Hi-0}}$ locus was delineated using 352 susceptible individuals. The number of recombinant individuals identified are are shown in bracket. Co-segregating markers are shown in red.

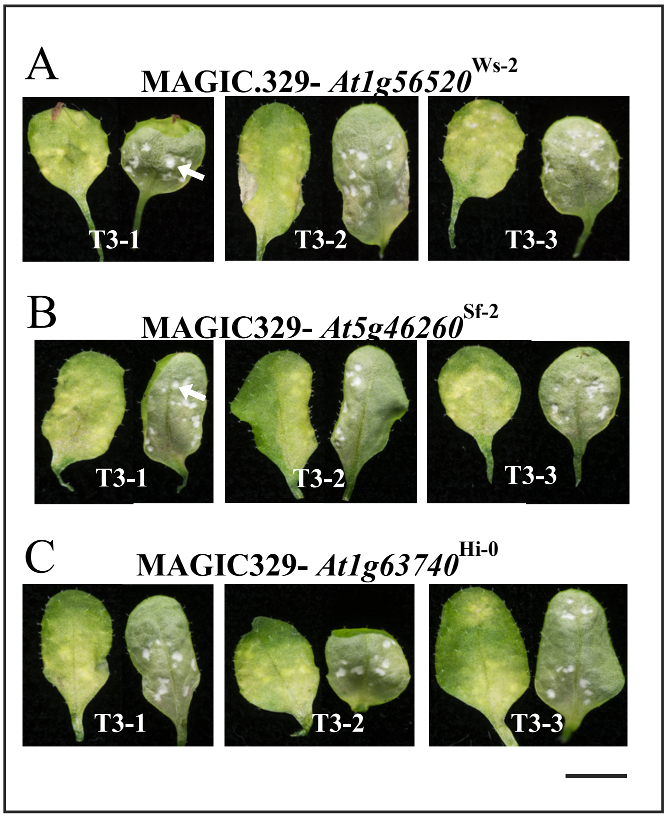

Fig S3. Paralogs of Distinct *WRR* genes do not confer resistance to Ac2V in transgenic MAGIC.329 lines. MAGIC.329 lines transformed with the genomic clones of (A) *WRR4B*$^{Ws-2}$ paralog *At1g56520*$^{Ws-2}$, (B) *WRR8*$^{Sf-2}$ paralog *At5g46260*$^{Sf-2}$, and (C) *WRR9*$^{Hi-0}$ paralog *At1g63740*$^{Hi-0}$. Interaction phenotypes were assayed in independent homozygous T_3 plants at 12 dpi. Examples of pustules (arrows) are indicated. Bar = 5 mm.

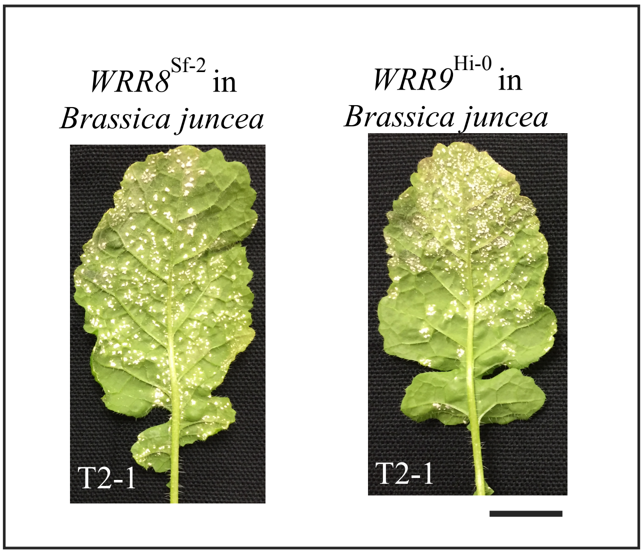

Fig S4. *Arabidopsis WRR8*$^{Sf-2}$ *and WRR9*$^{Hi-0}$ genes do not confer resistance to Ac2V in transgenic *Brassica juncea* plants . *Brassica juncea* plants were transformed with *WRR8*$^{Sf-2}$ or *WRR9*$^{Hi-0}$ and independent T_2 plants were tested with Ac2V and the pictures were taken at 15 dpi. Bar = 10 mm.

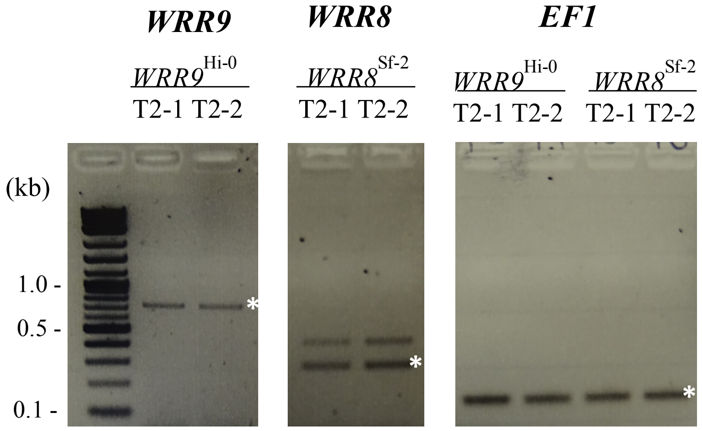

Fig S5. Expression analysis of *WRR8*$^{\text{Sf-2}}$, *WRR9*$^{\text{Hi-0}}$ and *Elongation Factor 1* (*EF1*) in transgenic *Brassica juncea* plants. Independent T_2 plants transformed with the indicated *WRR* genes were used for expression analyses. Expression analyses were carried out by reverse transcription polymerase chain reaction (RT-PCR). "*" indicates the spliced form of the gene.

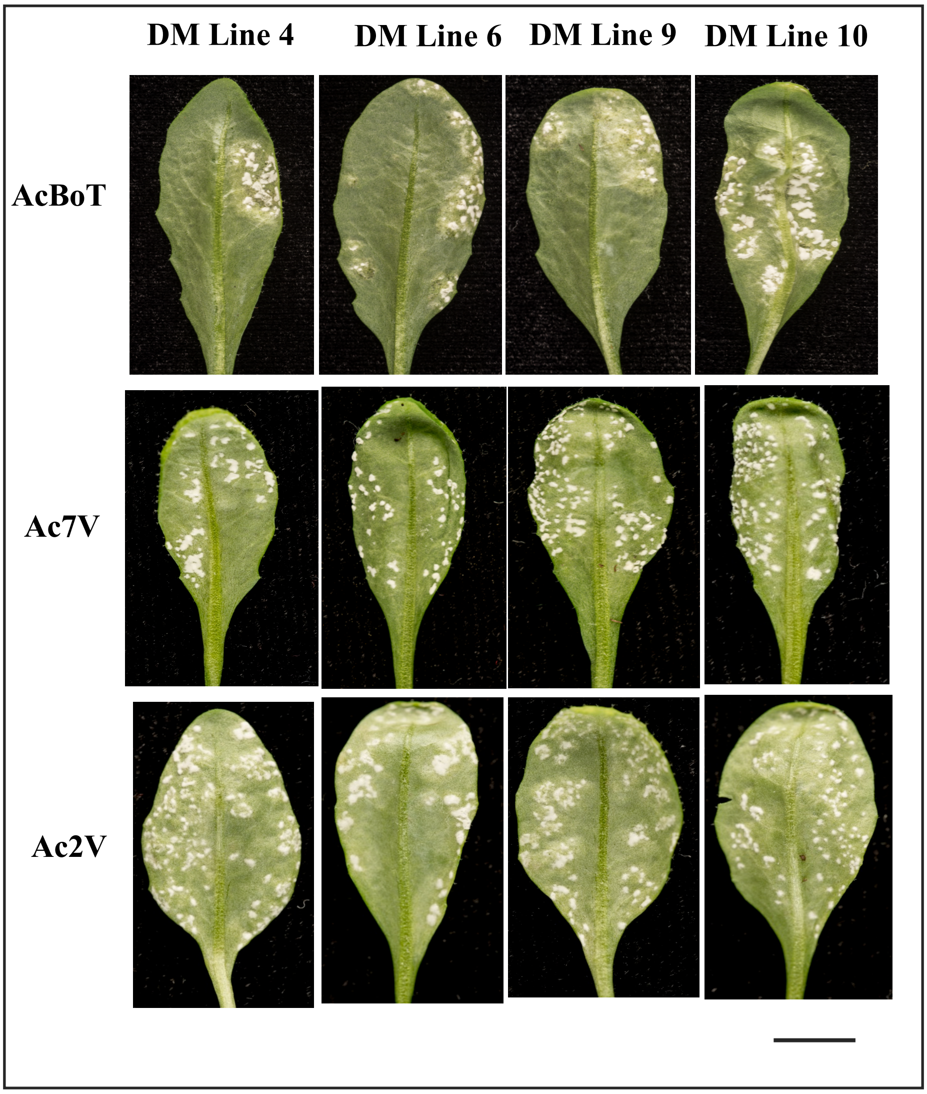

Fig S6. Independent 'Double MAGIC' (DM) lines susceptible to three different *Brassica*-infecting *A. candida* races. DM lines were inoculated with *B. oleracea*-infecting AcBoT, *B. rapa*-infecting Ac7V or *B. juncea*-infecting Ac2V *A. candida* races. Pictures were taken 15 dpi. Bar = 10 mm

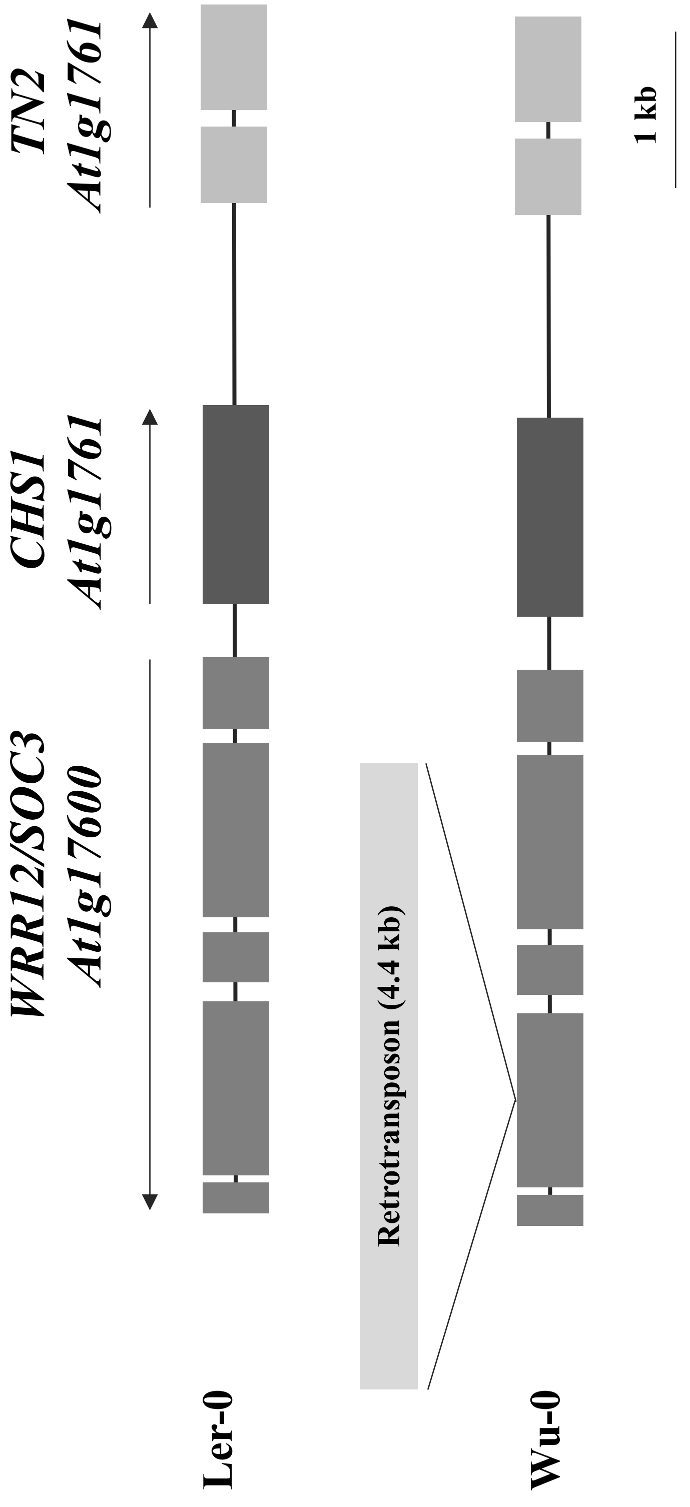

Fig S7. Genomic structure of the *WRR12* locus in MAGIC.329 and MAGIC.23. Exons and introns are shown as boxes and lines, respectively. Arrows indicate orientations of transcription. Location of the insertion of the Retrotransposon into MAGIC.23 allele of *At1g17600* is indicated.

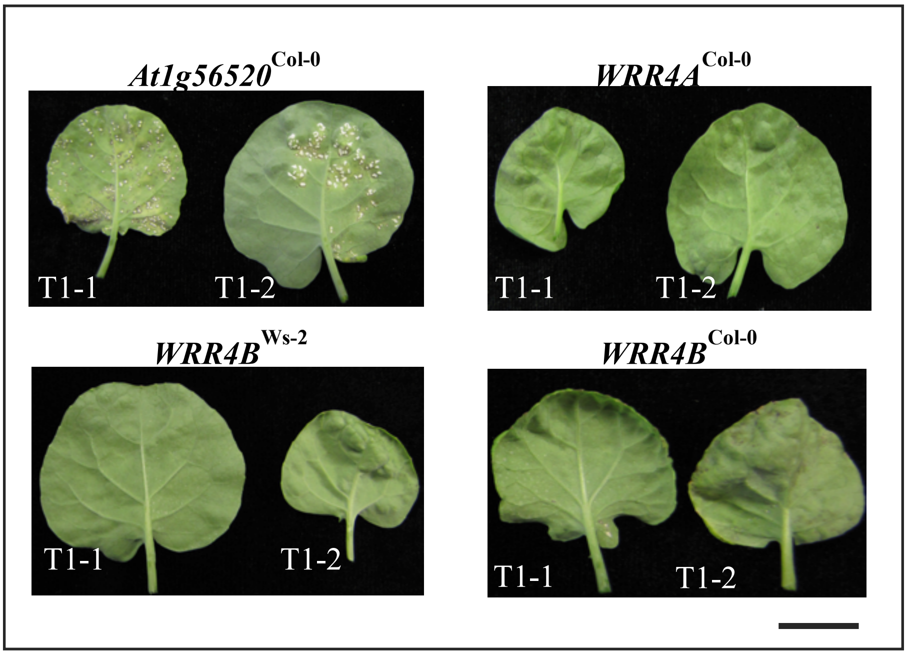

Fig S8. *Arabidopsis WRR* genes provide resistance to *Albugo candida* race AcBoT in *B. oleracea*. *Arabidopsis WRR4A*$^{Col-0}$, *WRR4B*$^{Ws-2}$, *WRR4B*$^{Col-0}$ and *At1g56520*$^{Col-0}$ as control were transformed in to *B. oleracea* and independent T_1 transgenic lines transformed with the indicated genes were inoculated with AcBoT and pictures were taken at 15 dpi. Bar = 10 mm.

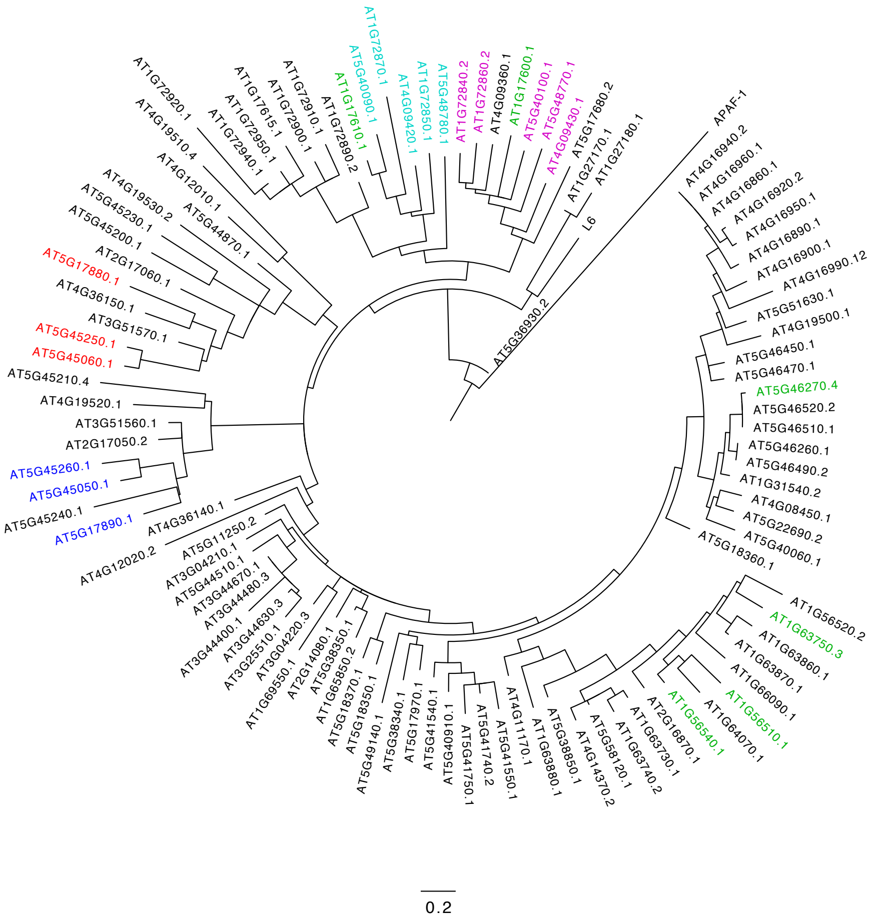

Fig S9. Phylogeny of *Arabidopsis thaliana* proteins containing TIR and NB domains. This phylogeny illustrates how the WRR12 TNL-TN pair are members of neighboring clades of TNL-TNs with the same paired structure, akin to the established head to head TNL pairs such as RPS4-RRS1. The Col-0 alleles of the White Rust Resistance proteins, WRR4 (AT1G56510), WRR4B (AT1G56540), WRR8 (AT5G46270), WRR9 (AT1G63750), WRR12 (AT1G17600 and AT1G17610) are colored green. The other WRR12-like pairs (TNL in genomic head to head conformation with TN) are colored in magenta (TNL) and cyan (TN). The known paired TNLs (RPS4-RRS1, RPS4B-RRS1B and CHS3-CSA1) from *Arabidopsis thaliana* are colored in red and blue. TIR-domain containing proteins were identified from the Araport11 Col-0 annotation using NLR-parser. Additionally, APAF-1 *(Homo sapiens)* and L6 (*Linum usitatissimum*) were chosen as outgroups. Where NB domains were detected, these were extracted and a core region of ~200 amino acids was aligned using MUSCLE. This alignment was used to build a maximum likelihood phylogeny with the WAG+F model. The scale bar refers to amino acid substitutions per site.

Table S1. Ac2V resistance in most MAGIC parents maps to the *WRR4* region.

Linkage analysis between Ac2V resistance and the *WRR4* locus using F_2 individuals derived from MAGIC.329 and the resistant *Arabidopsis* accessions was carried out.

Resistant parent	Linkage with the *WRR4* locus
Bur-0	YES
Can-0	YES
Col-0	YES
Ct-1	YES
Edi-0	YES
Hi-0	YES
Kn-0	YES
Ler-0	YES
Mt-0	YES
No-0	YES
Oy-0	YES
Po-0	YES
Rsch-4	YES
Sf-2	NO
Tsu-0	YES
Wil-2	NO
Ws-0	YES
Ws-2	YES
Wu-0	NT
Zu-0	YES

NT, not tested

Table S2. Identification of Linkage to the *WRR12*$^{MAGIC.329}$ locus.

			SNP (Frequency)		
Chr	AGI	Position	Ref (Col-0)	MAGIC.329	MAGIC.23
1	At1g12280	4176381	A	A (8.0%)	T (92.0 %)
1	At1g12280	4176420	T	T (8.0%)	A (92.0%)
1	At1g12280	4176441	A	A (9.0%)	G (91.0%)
1	At1g12290	4180867	T	T (8.0%)	G (92.0%)
1	At1g12290	4180973	C	C (7.0%)	T (93.0%)
1	At1g12290	4181341	T	T (8.0%)	G (92.0%)
1	At1g15890	Deleted in the MAGIC.329 (Parent) Genome			
**1**	**At1g17600**	**6053614**	**G**	**G (2.0%)**	**A (98.0%)**
**1**	**At1g17600**	**6054059**	**G**	**A (0.5%)**	**G (99.5%)**
**1**	**At1g17600**	**6056138**	**T**	**T (2.0%)**	**G (98.0%)**
**1**	**At1g17600**	**6056141**	**T**	**T (0.5%)**	**G (99.5%)**
**1**	**At1g17600**	**6056546**	**T**	**T (0.5%)**	**A (99.5%)**
**1**	**At1g17610**	**6057903**	**T**	**T (1.0%)**	**C (99.0%)**
**1**	**At1g17615**	**6059674**	**G**	**G (2.0%)**	**A (98.0%)**
**1**	**At1g17615**	**6059988**	**C**	**C (0.4 %)**	**T (99.6 %)**
1	At1g27170	9434667	G	A (9.0%)	G (91.0%)
1	At1g27170	9437672	G	G (10%)	C (90.0%)
1	At1g31540 (*RAC1*)	11292514	T	T (16 %)	C (84.0%)
1	At1g31540 (*RAC1*)	11292558	C	C (19%)	T (81.0 %)

Table S3. Primer sequences used for cloning and RT-PCR

Primer Name	Primer Sequence
WRR4A (At1g56510)-F	TCAGGAATCTCATTAGGCCCTGG
WRR4A (At1g56510)-BamHI-R	CAGCCTCGGATCCGTCAATTCACTAGACGATGTCG
GG-At1g56520-Ws2-Fragment1-F	TTGGTCTCAGGAGATCCGGTGTATGACTAGTCGGAC
GG-At1g56520-Ws2-Fragment1-R	TTGGTCTCACGATCCTTTGATCATCGAACATTG
GG-At1g56520-Ws2-Fragment2-F	TTGGTCTCAGATGCAGGCAATTCTTGTACC
GG-At1g56520-Ws2-Fragment2-R	TTGGTCTCAAGTCTCTTTATGATTTTTTCTCATG
GG-At1g56520-Ws2-Fragment3-F	TTGGTCTCAGACTATTCCTCTGTTTCCTTGC
GG-At1g56520-Ws2-Fragment3-R	TTGGTCTCAAGCGCATCACAGATTCCCAGAGAGCGTG
GG-At1g56540-Ws2-Fragment1-F	TTGGTCTCAGGAGGAGAGATGATCTTGAGTGGTTC
GG-At1g56540-Ws2-Fragment1-R	TTGGTCTCACGATCCTTTGATCATCGAACATTG
GG-At1g56540-Ws2-Fragment2-F	TTGGTCTCAATCGAGAGAATCCAAATCATCGC
GG-At1g56540-Ws2-Fragment2-R	TTGGTCTCAGAGTTCCTTCAAACGAGATGACC
GG-At1g56540-Ws2-Fragment3-F	TTGGTCTCAACTCCCAAATCTTTCAAATGCTA
GG-At1g56540-Ws2-Fragment3-R	TTGGTCTCAAGCGCTAGAGAGGACTTCTCCTTACG
GG-At1g56520-Col0-Fragment1-F	TTGGTCTCAGGAGCGAATCCACAGGACAATTATTAGC
GG-At1g56520-Col0-Fragment1-R	TTGGTCTCATATGAACAAATAGCAACTATTCC
GG-At1g56520-Col0-Fragment2-F	TTGGTCTCACATAGAATGCATATATGGACA
GG-At1g56520-Col0-Fragment2-R	TTGGTCTCACATTGCACTGGTGTTACTACTC
GG-At1g56520-Col0-Fragment3-F	TTGGTCTCAAATGCCTAAATGATGCAGGCA
GG-At1g56520-Col0-Fragment3-R	TTGGTCTCAGTCTCTTTATGATTTTTTCTCATG
GG-At1g56520-Col0-Fragment4-F	TTGGTCTCAAGACTATTCCTCTGTTTCCTTGC
GG-At1g56520-Col0-Fragment4-R	TTGGTCTCAAGCGCGAACGGATCCTATACGTCTACAC
GG-At1g56540-Col0-Fragment1-F	TTGGTCTCAGGAGGTCGCCGGAATCACCGTCGC
GG-At1g56540-Col0-Fragment1-R	TTGGTCTCACTGCAGGACCAGAGATTCCAAC
GG-At1g56540-Col0-Fragment2-F	TTGGTCTCAGCAGGCATTGGTAAGAGTACC
GG-At1g56540-Col0-Fragment2-R	TTGGTCTCAGAGTTGGAATGACTTGTAGACG
GG-At1g56540-Col0-Fragment3-F	TTGGTCTCAACTCTCACCAACTTGGTATCTCTC
GG-At1g56540-Col0-Fragment3-R	TTGGTCTCAAGCGAGTTACCCTAGAGAGGACTTCTC
USER-At5g46260-SF2-F	GGCTTAAUGACTCAAGAAACCTATGACTTGC
USER-At5g46260-SF2-R	GGTTTAAUCATCTACTCAAACTAGTCATGAAG
USER-At5g46270-SF2-F	GGCTTAAUGGCAAAGTTCATATCATTTGATC
USER-At5g46270-SF2-R	GGTTTAAUTAGAGGAGAAATAGTGAGGCGTCGCGTC
USER-At1g63730-Hi0-F	GGCTTAAUCAAGAGTCATAGATTAGGTCTAAGAC
USER-At1g63730-Hi0-R	GGTTTAAUGAAAATTGCCCAAATCGTATTGAG
USER-At1g63740-Hi0-F	GGCTTAAUGGTATCCAGATCTTTCACGGACACTAC
USER-At1g63740-Hi0-R	GGTTTAAUGGTGCTGCTGAATCTAGTACGTA
USER-At1g63750-Hi0-F	GGCTTAAUGCAGCAGTTCCAAGTGAATACTAG
USER-At1g63750-Hi0-R	GGTTTAAUCCGAGTCCATATCGTGGGCATCGAG
USER-At1g17600-Ler0-F	GGCTTAAUCAATGCTTTCATGTGACGGTCCATTC
USER-At1g17600-Ler0-R	GGTTTAAUTCCATGTGACTCAGAGGCAAC
B.juncea_EF1_RT-F	CCAAGAATGGGCTTTATGC
B.juncea_EF1_RT-R	GTGATAGAGTGTCCAACAAGGTAAGTA
At1g63750_RT-F	GCCCGACGTCCGTAAAACATTACTC
At1g63750_RT-R	GCAACTGCTGACCGTAATCGTGAA
At5g46270_RT-F	CGCTCAAAATGCCGAATAGCAAG
At5g46270_RT-R	CCGAGCAATATCGGAAGTTGAGG

Supplementary Dataset Legends

Dataset S1. Molecular markers and the primers used in this study.

Dataset S2. The distribution of WRR gene and protein alleles according to SMRT RenSeq on the MAGIC parents and Ws-2. A summary of the results from nucleotide (megablast) and protein (blastp) searches of the cloned WRR sequences against databases resulting from assembly and gene prediction from RenSeq reads, followed by a detailed table for each of the WRRs, including the amino acid sequence encoded by each predicted allele from the *de novo* assemblies.

Dataset S3. The sequences of the RNA oligos (baits) used to enrich sequencing libraries for NLR- encoding genes. A list of individual bait sequences and a table indicating the totals from each Brassicaceae species.
